# Adherence to dual antiplatelet therapy after coronary stenting: A study conducted at two Vietnamese hospitals

**DOI:** 10.34172/jcvtr.2021.52

**Published:** 2021-12-05

**Authors:** Anh Tien Hoang, Thi Quynh Nhu Tran, Cao Phuong Duy Le, Thi Ha Vo

**Affiliations:** ^1^PCI Unit, Hue University Hospital, Hue, 52000, Vietnam; ^2^Department of Internal Medicine, University of Medicine and Pharmacy, Hue University, Hue, 52000, Vietnam; ^3^Department of Pharmacy, University of Medicine and Pharmacy, Hue University, Hue, 52000, Vietnam; ^4^PCI Unit, Nguyen Tri Phuong Hospital, Ho Chi Minh, V-70000, Vietnam; ^5^Faculty of Pharmacy, Pham Ngoc Thach University of Medicine, Ho Chi Minh, V-70000, Vietnam; ^6^Department of Pharmacy, Nguyen Tri Phuong Hospital, Ho Chi Minh, V-70000, Vietnam

**Keywords:** Dual Antiplatelet Therapy, Acute Coronary Syndrome, Adherence, Knowledge

## Abstract

***Introduction:*** Adherence to dual antiplatelet therapy (DAPT) is critical after drug-eluting stent(DES) placement. We aimed to assess patient’s knowledge, rates of DAPT adherence, trends in DAPT use over time, and patient‐level factors associated with nonadherence in the patient with acute coronary syndrome (ACS).

***Methods:*** ACS patients who received one or more DES between May and September 2018from two hospitals in Vietnam and used DAPT after stent placement were eligible for a direct interview to assess patient’s knowledge on disease and DAPT. Telephone interviews were conducted one, three, and six months following discharge. Nonadherence was defined as premature discontinuation of DAPT. Factors related to nonadherent patients were analyzed using the chi-square test.

***Results:*** Of the 200 patients identified, 154 (77%) participated. Of the ten questions related to knowledge, the mean score of correct answers was 8.2 ± 2.3, and 71.7% had good knowledge.Adherence to DAPT was high at one month (94.2%) but declined by three months (44.2%) and then by six months (46.8%). Aspirin adherence was 99.3%-100% throughout. Three factors associated with nonadherence of DAPT following DES placement by six months included: rural location, linactive occupation, and inadequate knowledge on disease and DAPT (*p* <0.05).

***Conclusion:*** DAPT adherence is high at one month but is suboptimal at three and six months.Factors associated with the nonadherence of DAPT will be helpful in the planning of patient education strategies.

## Introduction

Over the past three decades, the treatment of percutaneous coronary intervention (PCI) in patients with coronary artery disease has been widely disseminated, contributing to significant improvements in the treatment of coronary artery disease. The administration of both aspirin and additional inhibitors of the platelet receptor P2Y12 (dual antiplatelet therapy [DAPT]), for a minimum of 12 months after DES placement unless there are contraindications such as the excessive risk of bleeding is recommended to prevent thrombosis by the ESC^1^ and ACC/AHA practice guidelines.^2^

However, noncompliance with antiplatelet therapy after coronary stenting is common^3^. Early discontinuation of therapy is the most powerful predictor of cardiac hospitalization and mortality for patients treated with a drug‐eluting stent (DES).^4^ Several factors associated with nonadherence to antiplatelet therapy have been examined by prior studies.^3^ Although numerous prior studies have been conducted in other countries, there has been little research on adherence to DAPT among patients after DES in Vietnam.^5^ Therefore, we aimed to assess the patient’s knowledge, the rates of DAPT adherence, the trends in DAPT using, and factors associated with nonadherence.

## Materials and Methods

### Research design and location

Patients over 18 years of age who received one or more DES between May 1, 2018, and September 30, 2018, from two hospitals (Hue Central Hospital and Hue University Hospital, in Hue, Vietnam) used DAPT after discharge were eligible for a direct interview. Patients were excluded from the study if they declined to participate, had an associated health condition such as neurological disease, dementia, or other mental health problems, were not able to be reached via telephone after three attempts.

### Measurement instruments

The patient information was collected through a direct interview by a questionnaire with one pharmacist (Tran T.Q.N.). The questionnaire consisted of socio-demographic and medical backgrounds of patients, and knowledge of disease and treatment, and adherence. The questionnaire was composed after a review of similar questionnaires used in other studies. They were modified by the author (Tran T.Q.N.) and underwent content validation by a peer group comprising two senior clinical pharmacists (Tran T.Q.N. and Vo T. H.) and one cardiologist (Hoang A.T.). Experts reviewed whether the questionnaire was appropriate with objectives and item generation through literature review. It was pre-tested on a group of 3 patients to ensure that the questionnaire was unambiguous. From the feedbacks of piloting, some questions were worded differently or placed in the best order. It comprised the following variables.

### Socio-demographic and clinical characteristics form

The socio-demographic data (such as age, gender, location, level of education, occupation) and comorbidity were collected by interviewing patient and double-checking the medical records.

### Development of knowledge questionnaire

The final questionnaire had a total of ten questions related to knowledge of coronary artery disease and antiplatelet therapy. An example of questions covering knowledge was “What are adverse effects of antiplatelet therapy?” Answers were provided with multiple choices and “Don’t know” followed by correct and incorrect responses to further evaluate the responses. One point was offered for each correct response and the total score was calculated. Score ranges of 0–7, 8-10 were considered as poor and good knowledge, respectively. We also asked patients two general questions: “Which level did they understand the doctors’ explanation: understand ≥50%, understand < 50% of information provided?, and “Do you have difficulty in adherence to antiplatelet therapy after discharge?”).

### Endpoints

The primary endpoints in this study were adherence rates and the identifying predictors of nonadherence of DAPT following DES placement. Adherence rate was defined as the percentage of prescribed doses that the patient reported taking over the 1-, 3- or 6-month period. Patients were considered adherent if they reported taking 80% or more of the medication during the prescribed time. Nonadherence was defined as premature discontinuation of DAPT or, on average, two or more missed doses per week^6^. Telephone interviews on DAPT discontinuation were conducted one, three-, and six-months following discharge. We then compared patients’ profiles between those who stopped and those who continued DAPT 6 months after DES treatment.

### Statistical analysis

All data were analyzed by using SPSS software version 20.0. All data were expressed in frequency, percentage, mean ± SD after checking that all data were normally distributed. The percentage of prescribed doses for the patient was calculated by multiplying the number of estimated missed doses per week by the total number of weeks in the course of therapy and subtracting this number from the number of doses prescribed in the 3- or 6-month period. The resulting number was then divided by the total number of doses prescribed and multiplied by 100 to achieve a percentage. Chi-squared test was used for intergroup comparison of categorical variables between responses by adherent and nonadherent patients. All analyses were two-tailed. The P-value of < 0.05 was statistically significant.

## Results

### Patient characteristics and clinical profile of participants

A total of 200 patients were identified, and 154 (77%) participated in the telephone questionnaire. The remaining 56 patients were excluded because one (6%) had died at the time of contact, 30 (25%) refused participation, 15 (7.5%) had invalid phone numbers, and 10 (5%) were not reached by the third telephone call. Patient responses are presented in Table 1.

**Table 1:** Baseline data of participants (n = 154)

**Profile**	**Number (N)**	**Percentage (%)**
Age (year)	≤ 60	50	32.5
	> 60	104	67.5
Gender	Male	95	61.7
	Female	59	38.3
Education	High school and lower	135	87.7
	Graduate or higher	19	12.3
Occupation	Inactive	59	38.3
	Active	95	61.7
Location	Urban	32	20.8
	Rural	122	79.2
Comorbidity	148	96.1
Re-examination on time	144	93.5
Insurance cover	150	97.4

The majority (61.7%) were male and less than 60 years old (67.5%). Regarding the educational level, only 12.3% had a higher educational level than high school. About fourth-five of the patient (79.2%) lived in urban areas. The employed people (61.7%) were the predominant group, and 96.1% had comorbidity.

### Knowledge regarding coronary artery disease and treatment

Of the ten questions related to knowledge, the mean score of right answers was 8.2 ± 2.3 compared to the maximum score of 11 and 71.7% had good knowledge. Frequency distributions of correct responses regarding knowledge were presented in Table 2. A high proportion of patients (90.9-100.0%) knew that their CAD was a severe disease that needed to be treated by surgery and drug therapy. All patients knew well information about their PCI, including the number of stents, type of stent, and number of PCIs. About 90% of patients knew the therapeutic effects and adverse drug reactions of antiplatelet therapy.

**Table 2:** Frequency distribution of correct response regarding knowledge

**Questions**	**Answers**	**N (%)**
**A. Questions to assess knowledge about coronary artery disease**		
A1. Do you know that you get a coronary artery disease (CAD)?	Yes	144 (93.5%)
A2. Do you know how to treat your CAD?	Yes (Intervention/surgery and drug therapy)	140 (90.9%)
A3. Do you know that coronary artery disease is a serious disease?	Yes	154 (100.0%)
**B. Questions to assess knowledge on PCI**		
B4. Do you know how many stents you have?	Yes (1, 2, 3, others)	154 (100.0%)
B5. Do you know which type of stent you have?	Yes (bare stent, DES)	154 (100.0%)
B6. Do you know how many times you have stent placements?	Yes (First, the second time, others)	154 (100.0%)
**C. Questions to assess knowledge of antiplatelet therapy**		
C7. Do you know which drugs should be taken after stent placement?	Yes (anticoagulants, antiplatelets, blood thinners, antithrombotic)	142 (92.2%)
C8. Do you know how long you must take dual antiplatelet therapy?	Yes (several months until physicians prescribe, at least 6 months, can last for 12 months)	74 (48.1%)
C9. Do you know what antiplatelet therapy is used for?	Yes (prevent stent occlusion/bridging occlusion/blood clot formation/stroke/others)	145 (94.2%)
C10. Do you know what adverse effects of antiplatelet therapy are?	Yes (Risk of bleeding/others)	138 (89.6%)
Total	Good (8-10 answers are “Yes”)	112 (72.7%)
	Bad (Have at least three questions “I don’t know” or wrong answer)	42 (27.3%)
	Mean + SD	8.2 ± 2.3 (max 10, min 6)
**D. General questions**		
D11. Understood poorly the doctors’ explanation at discharge	Understand less than 50% of the information given	15 (9.7%)
D12. Do you have difficulty in adherence to antiplatelet therapy?	Yes	29 (18.8%)

### Adherence of DAPT following DES placement

Adherence to DAPT was high at one month (94.2%) but declined by three months (44.2%) and then by six months (46.8%). Aspirin adherence was 99.3%-100% throughout (Table 3).

**Table 3:** Adherence of DAPT following DES placement

**Drug**		**At discharge**	**1 month**	**3 months**	**6 months**
No antiplatelet		0	0	1 (0.7%)	1 (0.7%)
Monotherapy	Aspirin	0 (0.0%)	9 (5.8%)	85 (55.2%)	81 (52.6%)
DAPT	Aspirin + Ticagrelor	54 (35.1%)	54 (35.1%)	1 (0.7%)	0 (0.0%)
	Aspirin + Clopidogrel	100 (64.9%)	91 (59.1%)	67 (43.5%)	72 (46.8%)
	Total of DAPT	154 (100.0%)	145 (94.2%)	68 (44.2%)	72 (46.8%)

### Predictors of nonadherence of DAPT following DES placement by 6 months

Factors associated with nonadherence of DAPT following DES placement by six months included: rural location, inactive occupation, and poor knowledge on disease and treatment (*P* < 0.05)** (**Table 4).

**Table 4:** Predictors of nonadherence of DAPT following DES placement by 6 months

**Parameters**		**adherence (n=72)**	**nonadherence (n=82)**	***P***
Age (year)	≤ 60 (n = 50)	29 (40.3%)	21 (25.6%)	0.052
	> 60 (n = 104)	43 (59.7%)	61 (74.4%)	
Gender	Male (n = 95)	41 (56.9)	54 (54.9)	0.257
	Female (n = 59)	31 (43.1)	28 (34.1)	
Location	Urban (n = 32)	21 (29.2%)	11 (13.4%)	<0.016*
	Rural (n = 122)	51 (70.8%)	71 (86.6%)	
Education	High school and lower (n = 135)	60 (83.3%)	75 (91.5%)	<0.126
	Graduate and higher (n = 19)	12 (16.7%)	7 (8.5%)	
Occupation	Inactive (n = 59)	14 (19.4%)	45 (54.9%)	6.39E-06*
	Active (n = 95)	58 (80.6%)	37 (45.1%)	
Knowledge	Good (n = 112)	61 (84.7%)	51 (62.2%)	0.002*
	Bad (n = 42)	11 (15.3%)	31 (37.8%)	
Understood poorly the physician’ counseling	Good (n = 19)	11 (15.3%)	8 (9.8%)	0.299
	Bad (n = 135)	61 (84.7%)	74 (90.2%)	
Having difficulty in adherence to antiplatelet therapy	Yes (n = 29)	10 (13.9%)	19 (23.2%)	0.142
	No (n = 125)	62 (86.1%)	63 (76.8%)	

*P < 0.05

## Discussion

### Knowledge regarding coronary artery disease, coronary stents, and antiplatelet therapy

Few studies that investigated patients’ knowledge about cardiovascular disease risk, prevention, disease and treatment in patients with CAD^7,8^. We found no study which examined the patient’s knowledge about coronary stents and antiplatelet therapy in patients after DES placement. Our study is the first one which determined knowledge of patients with DES and DAPT about their disease and treatment. 27.3% of patients had poor knowledge, of which patient had better knowledge of CAD and PCI than on antiplatelet therapy. Only 48.1% of patients knew the possible duration of DAPT. Future counseling at discharge should be focused on this issue.

### Adherence of DAPT following DES placement

Many studies found that DAPT adherence declines with increasing time after drug‐eluting stent implantation. Adherence to DAPT was high at 1 month (94.2%) but declined to 44.2% and 46.8% by 3 months and 6 months, respectively. According to a systematic review of 34 studies on adherence to DAPT after coronary stenting in 2014, the adherence rate was 85.6%-98.3% at 1 month, and 70.3%-98.8% at 6 months, and 42.8%-96.2% at 12 months.^3^ The study of Luu et al in Vietnam National Heart Institute in 2015 found that adherence to antiplatelet therapy after coronary intervention among patients with myocardial infarction was relatively high at 1 month; it begins to decline by 6 months, 12 months, and more than 12 months (less than 1 month was 90.29%; from 1 to 6 months 88.0%, from 6 to 12 months 75.43%, and after 12 months only 46.29% of patients).^5^ The rate of nonadherence to DAPT in our study was relatively low at 3 and 6 months, compared to previous studies. The optimal duration of DAPT in a given patient is determined by the balance between the individual risks of presenting a recurrent ischemic event or a hemorrhagic complication due to maintained antithrombotic treatment.^9^ Both guidelines^1,2^ recommend 12 months of DAPT with a P2Y12 inhibitor in addition to aspirin in ACS patients undergoing coronary stenting, with the possibility of shortening (6 months) in patients who are at high risk of bleeding. Therefore, the very low adherence rate of DAPT at 6 months alerted the need to target patients who are more likely to nonadherence to solve problems.

A P2Y12 inhibitor includes prasugrel, ticagrelor, clopidogrel. In Vietnam, according to the Ministry of Health, the insurance covers only aspirin and clopidogrel^10^. The rate of insurance cover of patients in our study was 97.4%. Maybe insurance cover explains partly why aspirin and clopidogrel were prescribed as high as 64.9% while aspirin and ticagrelor were prescribed as low as 35.1% at discharge and declined to 46.8% and 0% by 6 months.

### Predictors of nonadherence of DAPT following DES placement by 6 months

Several factors associated with nonadherence to antiplatelet therapy have been found in prior studies. They include patient’s profile (lower education level,^4^ immigration status,^11^ ethnic minorities,^12^ elderly^13^), medical condition (previous major hemorrhage,^11^ chronic kidney disease^12^), patient’s perception (poor awareness of antiplatelet drugs, lack of understanding about medical conditions or the value of treatment adherence), drug-related reasons (side effects of drugs,^6^ ineffective treatment^6^), and financial barriers (income,^5^ high drug costs^6^ and financial reasons^6^).

In our study, rural location, inactive occupation, and wrong knowledge on disease and treatment, were factors associated with nonadherence of DAPT following DES placement by 6 months. There were 79.2% of patients who were likely to be nonadherent, live in a rural area. Maybe the reason for living in a rural area had less chance to access medical services. The study of Luu et al showed that adherence to antiplatelet treatment after the coronary intervention was significantly related to the distance from home to the hospital.^5^ This analysis clarified that wrong knowledge on disease and treatment plays a critical role in nonadherence to therapy; therefore clearly communicated instructions regarding disease and DAPT before discharge should be implemented. In our study, we asked two general questions which we predict that two answers to these two questions would be predictors of nonadherence. However, both answers (poor understanding of physician’s counseling and having difficulty in adherence to dual antiplatelet therapy) were not factors related to nonadherence to DAPT at 6 months. These findings suggest that these two simple questions cannot be helpful to target patients who would be likely to be nonadherent.

In 2013, the Working Group of Exercise Rehabilitation and Sport (GERS) and the Therapeutic Education Commission of the French Society of Cardiology issued a paper position on Therapeutic patient education (TPE) in coronary heart disease.^14^ Our study can suggest which knowledge patients lacked to shape the patient education and target specific patients for education to improve adherence. The patients received an adequate explanation in the hospital before they were discharged, and they again acquired the adherence when they came for re-examination or patient-centered tablet application^15^ or telephone contact.^16^

Our study is one of the first ones which assessed knowledge in patients after DES used DAPT and the first study determined the accurate adherence of DAPT in the patient after DES placement in the two most prominent hospitals in the Central Region of Vietnam. This study had several limitations. Firstly, because of the high rate of patients who refused to participate in or were not able to contact by phone three consecutive times, the sample size was not as large as expected. Most of the participants were patients who were willing to participate in the study, which may not be representative of the entire target population of patients at the two hospitals.

## Conclusion

DAPT adherence is high at 1 month but is suboptimal at 3 and 6 months. Factors associated with the nonadherence of DAPT will be helpful in the planning of patient education strategies.

## Acknowledgements

The authors wish to thank hospital clinical pharmacists and physicians in Hue University Hospital, Hue Central Hospital, and Nguyen Tri Phuong Hospital for helping us to conduct the study.

## Competing interests

The authors declare that they have no conflict of interests.

## Ethical approval

The study was approved by the local university ethical review board (Document Number: H2018/066). The study was conducted in a spirit of respecting the private information and patients had oral informed consent before data collection. After data collection, patients were encouraged to contact a healthcare professional if they voiced any concerns or adverse events regarding DAPT therapy.

## Funding

No funding.
